# The Impact of Implant Surface Modifications on the Osseointegration Process: An Overview

**DOI:** 10.7759/cureus.81576

**Published:** 2025-04-01

**Authors:** Bansi B Sarvaiya, Santosh Kumar, Mohd. Shabankhan H Pathan, Shirishkumar Patel, Vineeta Gupta, Mainul Haque

**Affiliations:** 1 Department of Periodontology and Implantology, Karnavati School of Dentistry, Karnavati University, Gandhinagar, IND; 2 Department of Periodontology and Implantology, Government Dental College, Chhattisgarh, Raipur, IND; 3 Department of Pharmacology and Therapeutics, National Defence University of Malaysia, Kuala Lumpur, MYS; 4 Department of Research, Karnavati School of Dentistry, Karnavati University, Gandhinagar, IND

**Keywords:** biological modifications, bone morphogenetic proteins, chemical modifications, dental implant, dental technology, implant surface characteristics, implant surface treatments, osseointegration, plasma coating, plasma-sprayed hydroxyapatite

## Abstract

Osseointegration is critical to the long-term success of endosseous dental implants. Surface factors such as roughness, topography, energy, and composition considerably impact this process. Several ways have been used to optimize surface roughness, increase surface area, and improve osseointegration. Subtractive processes such as alumina and titanium dioxide blasting, acid treatment, anodization, and laser peeling are widely utilized. Many additive techniques change implant surfaces, including plasma-sprayed hydroxyapatite, vacuum deposition, sol-gel, dip coating, electrolytic procedures, and nano-hydroxyapatite coating. Recently, biomimetic implant surfaces with calcium phosphate coatings have been created under physiological settings. These coatings can transport osteogenic agents such as bone morphogenetic proteins, growth differentiation factors, and bioactive medications, including bisphosphonates, gentamicin, and tetracycline. Advances in technology have considerably broadened the methods for surface modification of endosseous dental implants. This article provides a comprehensive overview of various surface modification techniques and current trends in oral implantology.

## Introduction and background

Dental implants have emerged as a reliable choice for restoring oral function in partially or entirely edentulous individuals, as they provide a strong basis for various types of prostheses [1,2]. Osseointegration, or the direct and intimate link formed between the implant and the surrounding bone, is a fundamental component of their success [1,3]. To enhance this process, surface engineering has gained increasing attention as a crucial and natural advancement in implant technology [4]. A key factor in surface engineering is the influence of surface wettability. Hydrophilic surfaces, in contrast to hydrophobic ones, exhibit enhanced interactions with biological fluids and cells, which may contribute to more effective osseointegration [4]. Moreover, the variety in implant designs plays a significant role in enhancing their performance. Today, there are around 1,300 distinct implant systems, differing in shape, dimensions, core and surface materials, thread patterns, implant-abutment connections, surface texture, chemical composition, wettability, and surface modifications [5]. Contact osteogenesis relies on specific stimuli to occur effectively among the factors that affect osseointegration. Surface modification of the implant is critical in speeding up this process, highlighting its importance in the development of implants [6]. Historically, improving the surface of a titanium implant has been associated with higher biocompatibility, greater bio-affinity with hard tissue, and a faster bone response [7]. The modern perspective on osseointegration suggests that hard tissue more readily recognizes a modified titanium surface, leading to a quicker and more robust accumulation of bone material to isolate the implant [7]. Consequently, ongoing research continues to explore the true nature of osseointegration and the factors that optimize its success.

Problem statement of this narrative review

Osseointegration is a critical factor in the long-term success of dental and orthopedic implants. The surface characteristics of an implant play a key role in influencing the biological response, directly impacting the rate and quality of osseointegration. Despite extensive research in this field, there is still a need to consolidate findings on how different surface modifications, e.g., roughness, coatings, and chemical treatments, affect the process. A comprehensive review of existing literature can clarify the most effective surface modifications, highlight gaps in current knowledge, and guide future research and clinical applications. Reviewing this topic will contribute to the ongoing discourse by summarizing advancements, comparing different modification techniques, and identifying potential areas for innovation in implant technology.

Objectives of this review paper

This study aims to provide a comprehensive review of existing literature on implant surface modifications and their impact on the osseointegration process. We planned to analyze and compare surface modification techniques, such as surface roughening, coatings, and chemical treatments, to determine their effectiveness in enhancing bone-implant integration.

## Review

Materials and methods

**Figure 1:**
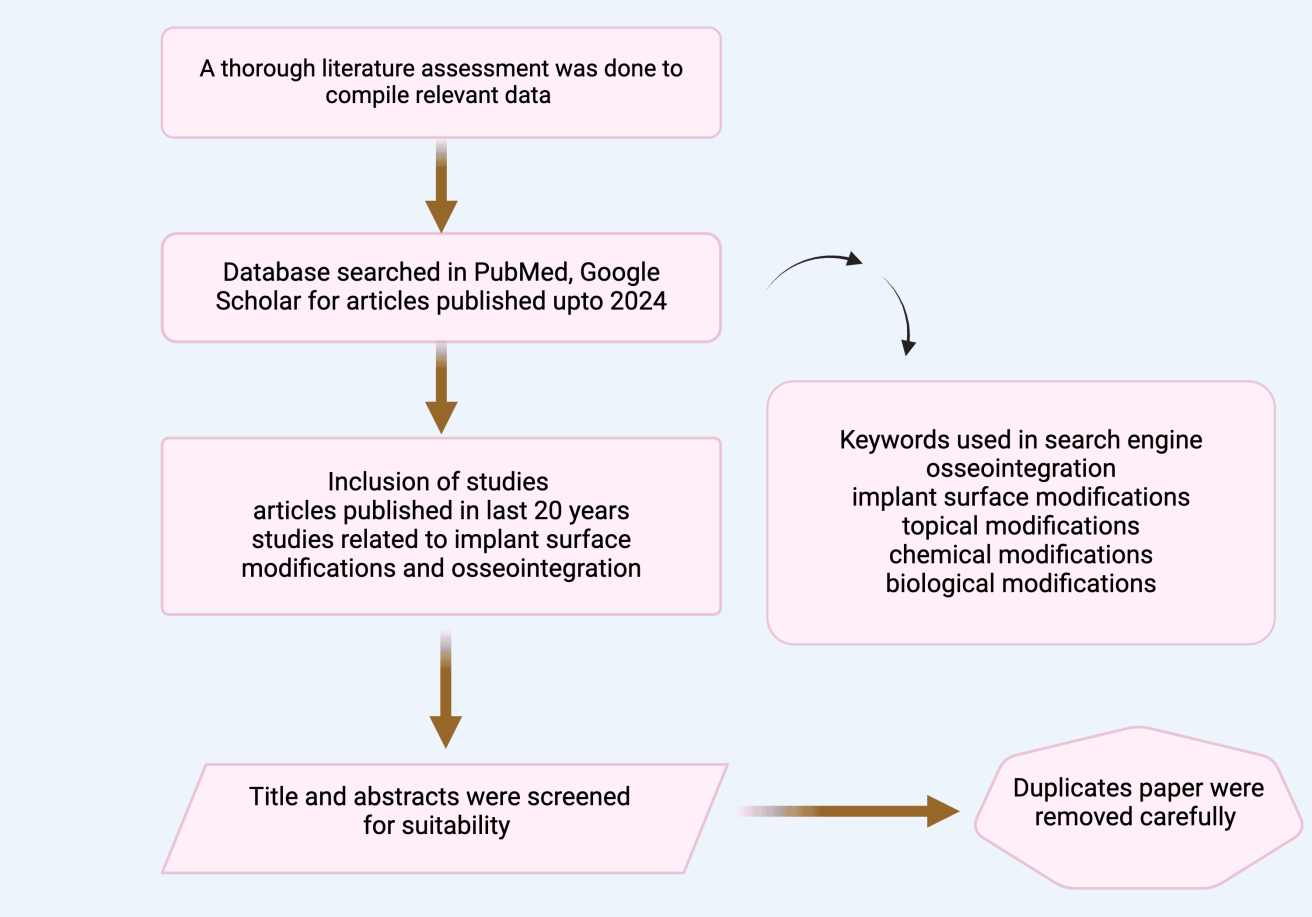
Study selection process for literature review on implant surface modifications and osseointegration. Note: This figure was drawn using the premium version of BioRender (https://BioRender.com/) [8], accessed on March 16^th^, 2025, with agreement license number ﻿RV2816SEEZ. Image credit: Bansi Sarvaiya.

Articles were searched using Google Scholar, PubMed, and Web of Science using keywords, including "Implant Surface Modification," AND "Osseointegration," AND "Sandblasting," AND "Grit Blasting," AND "Chemical Coatings," AND "Bioactive Surface Coatings", that improve osseointegration. Key insights were gleaned from the chosen publications, which provided vital information on surface modifications in dental implants (Figure 1).

**Figure 2:**
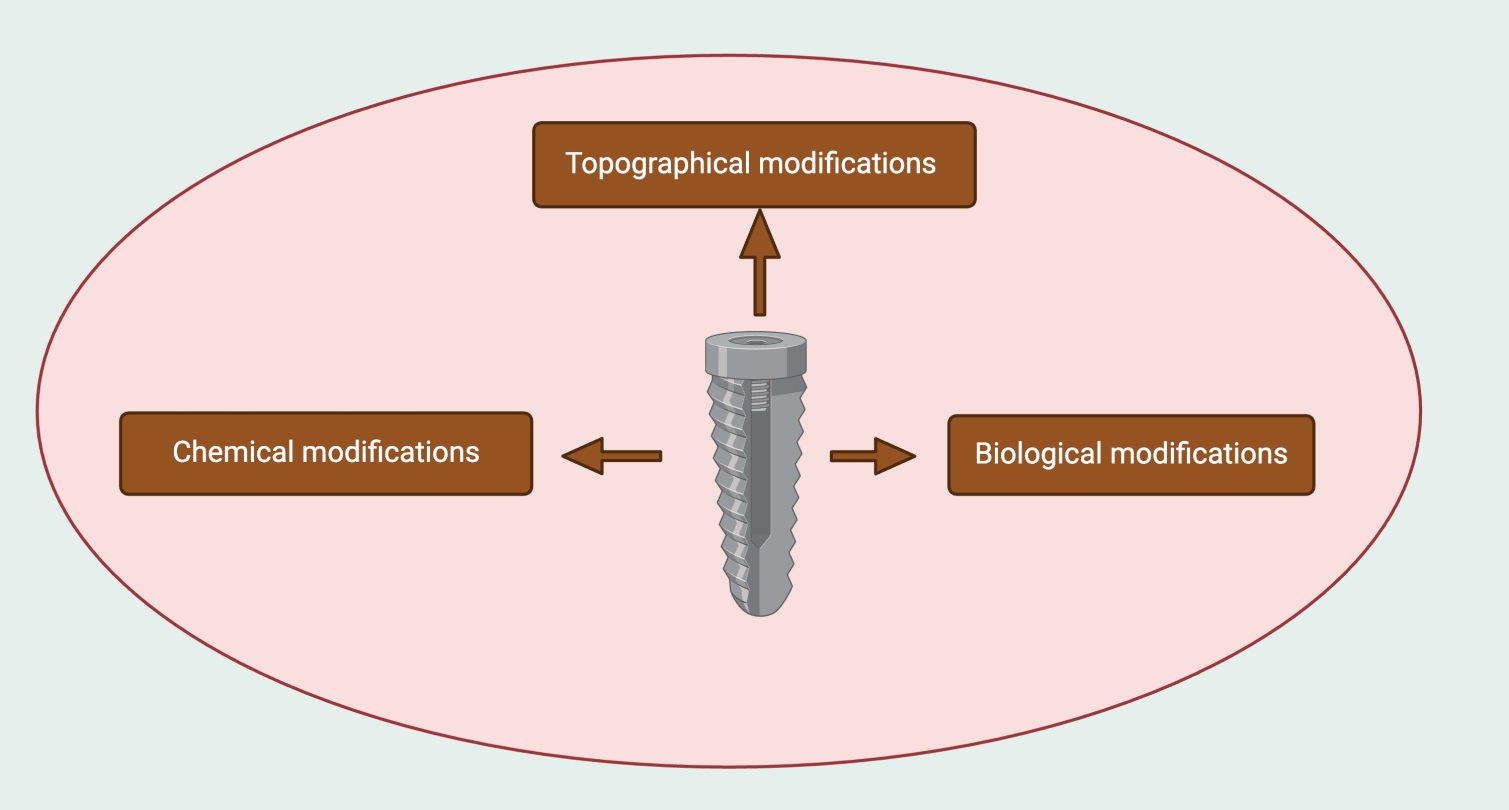
Overview of dental implant surface modification strategies. Note: This figure was drawn using the premium version of BioRender (https://BioRender.com/) [8], accessed on March 16th, 2025, with agreement license number O36K697. Image credit: Bansi Sarvaiya.

Surface alterations in dental implants are critical for promoting osseointegration, increasing implant durability, and ensuring long-term success. Several approaches have been developed to modify implant surfaces, maximizing their biological and mechanical qualities to improve bone integration. These alterations can be classified into topographical, chemical, and biological techniques (Figure 2).

Review of literature

Topographical Modification

These modifications change the surface roughness and microstructure of the implant to improve bone cell attachment and growth. Standard techniques include the following.

Sandblasting: It uses abrasive particles to create a roughened surface, improving mechanical interlocking with bone.

Acid etching: It creates micropores on the implant surface, increasing surface area and promoting cellular adhesion.

Laser treatment: It generates controlled micro- and nano-scale surface patterns that enhance osseointegration.

**Figure 3:**
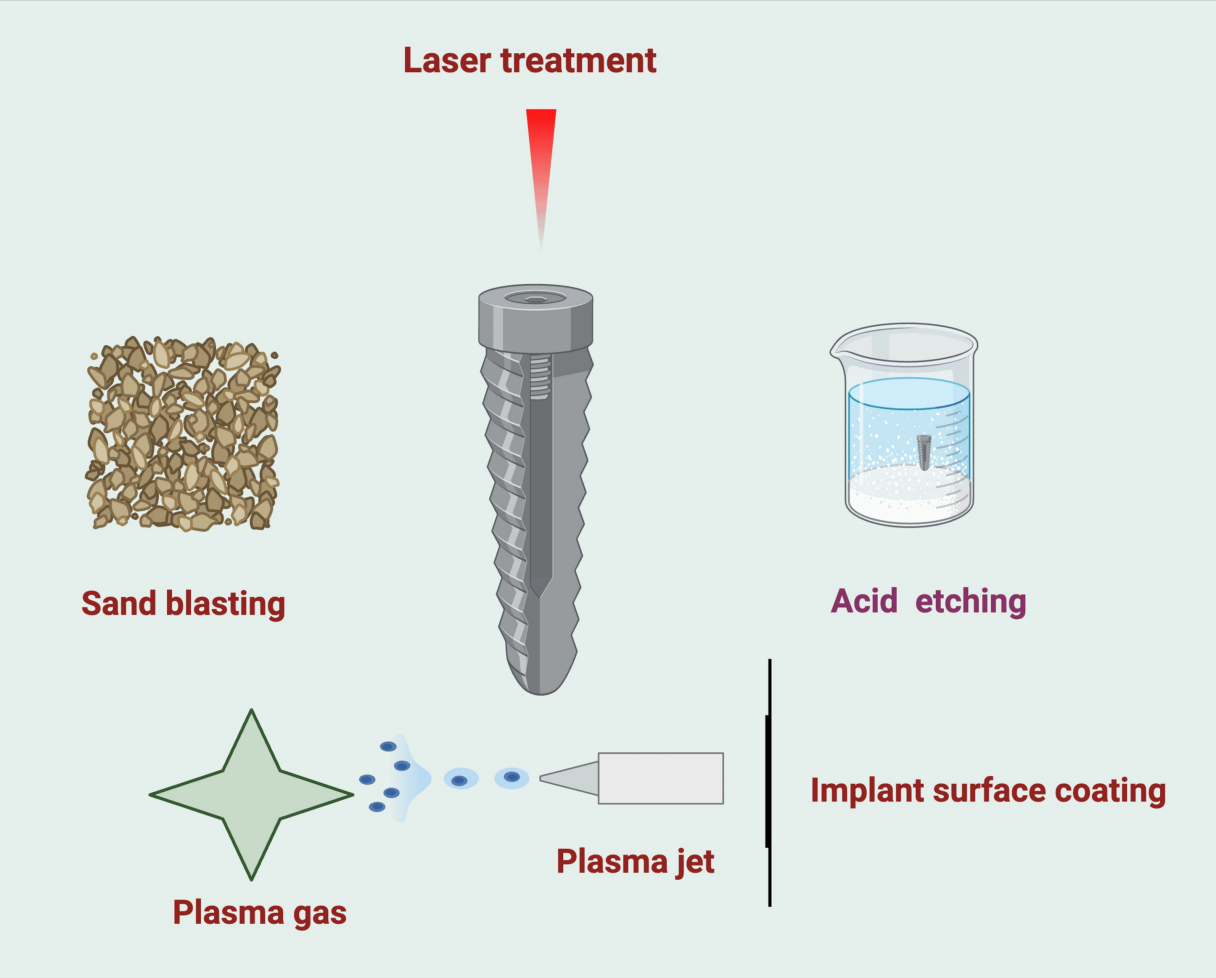
Techniques for dental implant surface modification. Note: This figure was drawn using the premium version of BioRender (https://BioRender.com/) [8], accessed on March 16th, 2025, with agreement license number U91E507. Image credit: Bansi Sarvaiya.

Plasma spraying: It deposits a layer of bioactive materials, such as hydroxyapatite, to promote bone bonding (Figure 3).

Chemical Modifications

Chemical treatments modify the surface chemistry of implants, enhancing their wettability and biocompatibility. These include the following.

Anodization: It forms a thick titanium oxide layer, increasing surface roughness and bioactivity.

Hydroxylation and fluorination: They alter surface composition to improve protein and cell interactions.

**Figure 4:**
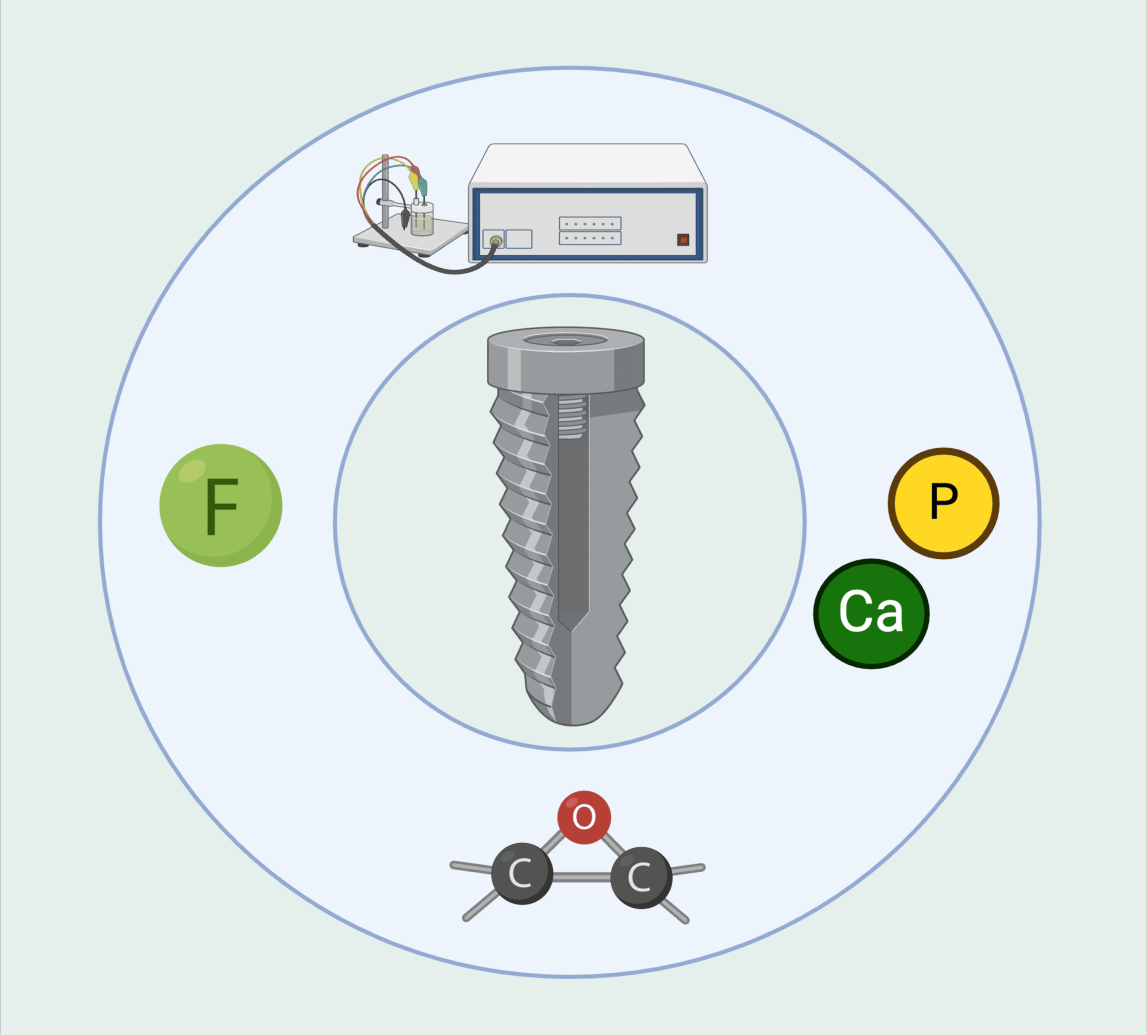
The image depicts chemical modification on the implant surface. Note: This figure was drawn using the premium version of BioRender (https://BioRender.com/) [8], accessed on March 16th, 2025, with agreement license number M80Z357. Image credit: Bansi Sarvaiya.

Silicon and calcium phosphate coating: It enhances osteoconductivity and accelerates bone formation (Figure 4).

Biological Modifications

These modifications involve incorporating bioactive molecules to stimulate bone healing and integration. These techniques include the following.

Growth factor coatings: These coatings utilize proteins like bone morphogenetic protein (BMP) to enhance osteogenesis.

Drug delivery coatings: These coatings release antibiotics or anti-inflammatory agents to prevent infections and promote healing.

**Figure 5:**
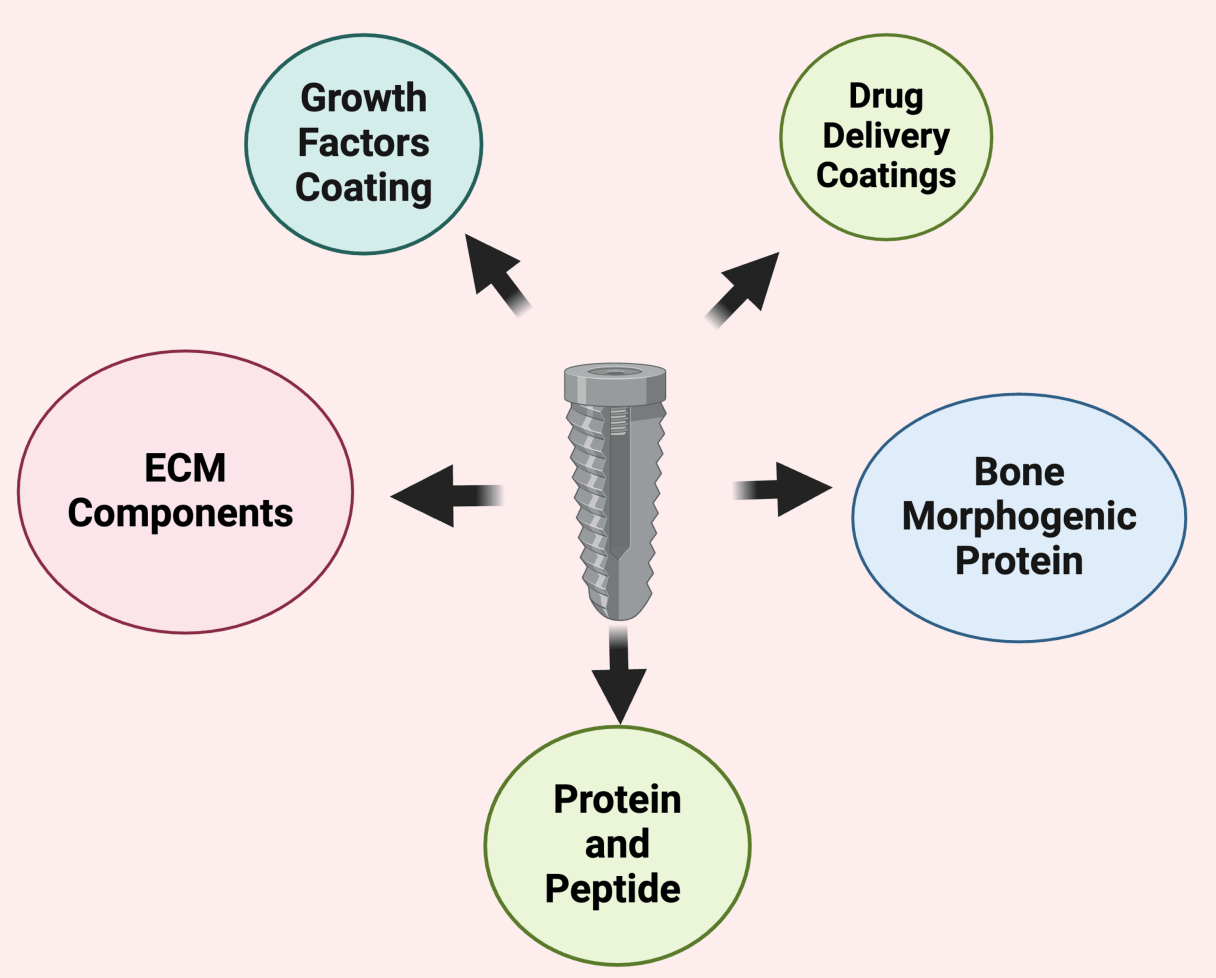
Schematic representation of bioactive coating strategies for dental implants. Note: This figure was drawn using the premium version of BioRender (https://BioRender.com/) [8], accessed on March 21^st^, 2025, with agreement license number OD281V3SGV. Image credit: Bansi Sarvaiya.

Peptide and protein functionalization: It improves cellular responses by mimicking natural extracellular environments (Figure 5).

Implant Surface Topography

Implant surface topography encompasses both macroscopic and microscopic aspects of the implant surface. While industrially available titanium (Ti) is the most frequently utilized material for implants in dentistry, success rates fluctuate amongst commercially accessible implant systems. Titanium implants with optimum surface roughness can influence essential stability, enhance bone-to-implant contact (BIC), and even increase removal torque [6].

Surface roughness expands the surface area and influences cell morphology, promoting osteoblastic differentiation, bone formation, and remodeling [2,4]. The primary objectives of implant surface modifications are to improve clinical performance in regions with limited bone quantity or quality, speed up bone healing to support immediate or early loading protocols, and promote bone growth. This, in turn, allows implant placement in sites with inadequate residual alveolar ridges, granting them the ability to bridge gaps effectively [9].

Topographical Modifications

Computer numerical control (CNC) machining of commercially pure titanium (cp-Ti) makes screw-shaped endosseous dental implants. When viewed under scanning electron microscopy, the surface produced by this milling process, known as a turned titanium (Ti) surface, shows multiple parallel grooves. Because the turned surface experiences no further alterations, it has frequently been employed as a control to examine the biocompatibility of various surface modifications [10]. Mechanical techniques were commonly used in early implant manufacturing systems until the 1990s. Surface enhancement methods such as shot peening (SP) and abrasion gradually refined the outer layer's particle size. Meanwhile, machining, grinding, refinishing, and sandblasting (SB) modified the surface layer and enhanced its roughness [11].

Grit blasting: Titanium dioxide particles (25-50 μm) are often blasted onto implants to increase surface roughness. Grit-blasted implants with roughened surfaces have a better success rate than machined implants. A 10-year clinical follow-up study discovered a 96.9% survival rate for titanium implants undergoing titanium dioxide grit-blasting, suggesting an improved prognosis than smooth polished surfaces, particularly in low-density bone. This surface modification leads to better long-term clinical results for both the upper jaw and mandible [12].

Sandblasting: This process involves exposing implants to high-pressure gritting agents such as alumina (Al₂O₃) and titanium oxide (TiO₂). The quantity and size of the particles, the applied pressure, and the implant's rotational speed all impact the process's success [13,14].

Acid etching: Strong acids can also be used to roughen the surface of titanium dental implants. This process eliminates the oxide layer and sections of the underlying material, with the degree of material removal determined by factors such as acid concentration, temperature, and exposure time. Mishra et al. (2018) [9] reported that commonly, titanium is etched using a solution composed of either nitric acid (HNO₃) and hydrofluoric acid (HF) or a combination of hydrochloric acid (HCl) and sulphuric acid (H₂SO₄) [9]. The etching rate is affected by the composition of the acid mixture, the etching duration, and the temperature of the bath [9]. Acid etching after shot-blasting improves hydrophobicity while increasing surface energy. These two factors, i.e., wettability, which promotes osseointegration, and energy, which inhibits it, counterbalance each other in bone development. Moreover, the roughness increase from acid etching on shot-blasted samples was minimal and did not significantly impact bone colonization [15].

Laser treatment: Recently, laser-based approaches for improving surface roughness have received much attention. Lasers provide accurate frequency regulation, a broad frequency range, high energy concentration, focused and mastered light, and the ability to pulse the source for regulated reaction times [16]. Laser polishing substantially increases the surface quality of 3D-printed polyether ether ketone (PEEK) biomimetic dental implants. The following discussion examines the significance of these findings and their relation to biomimetic dental implantology [17].

Plasma spraying: Plasma is a neutral-position charged gas with considerable potential energy. It consists of particles such as photons, electrons, positive and negative ions, atomic particles, free radicals, and energized and inactive molecules. It can be manufactured and used to treat surfaces in various ways [18]. Utilizing plasma under gas conditions minimizes hydrocarbon impurity accumulation on the implant surface [19]. The effect of atmospheric jet plasma on osteoblastic cells was analyzed in terms of contact angle, cell area, and metabolic activity. The contact angle was influenced by both exposure time (30, 60, and 120 seconds) and gas composition (Ar-plasma, 0.2% O₂/Ar-plasma, 1.0% O₂/Ar-plasma), but it remained unaffected by surface topography [20]. Plasma treatment improves adherence, particularly on hydrophilic surfaces. The roughness of titanium may impact the organization of the cytoskeleton, which affects the attachment of osteoblastic cells [21].

Chemical Modification of the Implant Surface

Several chemical changes have been applied to implant surfaces to improve osseointegration and minimize biofilm formation by creating a hydrophilic coating. The most commonly used processes are discrete crystallized accumulation, anodized oxidation, and ultraviolet (UV) light therapy, also known as photo functionalization. Chemically altered implant surfaces can significantly enhance bone healing [12].

Anodization: In anodic oxidation, the implant is an anode within an electric circuit. TiUnite implants have been shown to have nanoscale surface characteristics. Furthermore, cell experiment data indicate that anodic oxidation might be applied to the implant's neck to facilitate the creation of stable soft tissue [22]. Anodic electrochemical oxidation provides fine control over the characteristics of the oxide layer, which is heavily impacted by the electrochemical conditions used. At the ultrastructural level, the surface topography is determined by field-assisted ion migration inside an oxide film.

In contrast, the thickness of the anodic oxide layer is determined by Faraday's law. Anodization improves the texture of the titanium surface by forming a thick oxide layer of roughly 1500 nm. This layer predominantly comprises crystalline anatase TiO₂ and includes micro-pores and nano-tube arrays. The increased surface roughness, reduced water contact angle, and enhanced wettability produce a hydroxylated, hydrophilic surface, encouraging osseointegration [23].

Fluorination: Fluorine, a trace element required for numerous biological activities, is vital for bone formation and maintenance. Fluoride at the micromolar level can stimulate the activities of osteoblasts, raise alkaline phosphatase activity, and increase the bone apposition rate in the early stages of osteogenesis [24]. The use of fluoride, whether through subtraction [12,20], as in this work, or addition [16], is regarded as one of the most promising implant modifications. Regardless of the type of fluoride-treated implant surface, these surfaces have been proven to promote the osseointegration process in preclinical models [15,21] and have shown positive clinical outcomes with high survival and success rates [25].

Silicone and copper oxide coatings: Biocompatible implant materials with improved antibacterial qualities, osteogenic potential, and angiogenic capabilities aid in osseointegration. Microporous TiO₂ coatings (M-Ti) doped with Cu²⁺ (M-Cu) and varying concentrations of Si²⁺ (M-CuSi) were directly fabricated using a straightforward one-step micro-arc oxidation (MAO) process [26].

Calcium phosphate coatings: Ca^2+^-phosphates are commonly used in commercial and educational studies. These ceramic materials are chemically comparable to bone mineral, a naturally occurring element in human bones [21-27]. Within the body, their biological activity assures that metallic bone implants function well over time. They prevent bone anchor failure and delay the need for revision surgery [27]. Amorphous calcium phosphate (ACP), a different stage in the calcium orthophosphate group, is more effective than crystalline hydroxyapatite (HAP) in fostering early bone growth and remineralization [14]. From the "bio-reactivity" standpoint, ACP may facilitate bone-like apatite formation more efficiently, encouraging faster bone regeneration [28].

Biological Modifications

The additional benefit of sustained local release of bioactive chemicals supports bone tissue growth, enhancing the osseointegration possibility of titanium implants, hence reigniting the desire for bone tissue engineering.

Growth factor coatings: Growth factors are a class of bioactive substances with considerable osteogenic and angiogenic properties. Growth factors, such as BMPs, vascular endothelial growth factor (VEGF), platelet-derived growth factor (PDGF), transforming growth factor (TGF), growth factor analogous to insulin (IGF), hepatocyte growth factor (HGF), and fibroblast growth factor (FGF), serve an essential part in controlling various cellular processes such as differentiation, apoptotic morphological development, the development of embryos, blood vessel development, wound healing, hematopoietic regulation, and inflammation. Adding one or more growth hormones into the bone-implant contact can increase osteoblast activity, accelerate the osteogenic process, and stimulate bone formation [29].

Bone morphogenetic protein (BMP): BMP, a member of the transforming growth factor-beta (TGF-β) superfamily, regulates osteogenic cells and differentiates bone mesenchymal stem cells (MSCs), playing a vital role in osteogenesis [6,11]. BMPs are frequently used as an adjuvant for bone graft material; their inclusion contributes to bone-to-implant contact [30]. Using bone morphogenetic protein 7 (BMP7) coatings on dental implants enhances osseointegration [31].

Vascular endothelial growth factor (VEGF): VEGF-A plays a crucial role as a growth factor in controlling vascular development and the process of angiogenesis. Because bone is a highly vascular organ and angiogenesis is essential for osteogenesis, VEGF impacts skeletal development and postnatal bone repair, so it can also be used as a coating on dental implants [32]. Incorporating VEGF as a coating on dental implants enhances angiogenesis around implants, indirectly enhancing osteogenesis and leading to rapid osseointegration of implants [33].

Platelet-derived growth factors: Recombinant platelet-derived growth factor-BB (PDGF-BB) stimulates bone development around implants, which is promising for enhancing clinically guided bone regeneration (GBR) procedures. Recombinant human platelet-derived growth factor-BB (rhPDGF-BB) affects the earliest stages of osseointegration for dental titanium implants [34,35].

Transforming growth factors: Human recombinant transforming growth factor beta 1 (TGF-β1), a multifunctional regulatory protein, will achieve a faster and larger percentage surface of osseointegration [36]. The application of recombinant human transforming growth factor-beta 1 (rhTGF-β1) at implant sites seems to enhance the amount of bone healing near titanium dental implants [37].

Insulin-like growth factors: Insulin growth factor binding protein-3 (IGFBP-3) reduces glucose oxidative stress and stimulates bone formation [38]. The insulin-like growth factor boosts osteoblastic activity surrounding dental implants in type 2 diabetes patients [39,40].

Hepatocyte growth factors: It was discovered that hepatocyte growth factor (HGF) can be bound to the hydroxyapatite surface of implants, increasing bone formation and angiogenesis activities throughout the osseointegration process [41]. Awad et al. (2021) reported increased osteoblast proliferation when cells are grown on HGF-coated hydroxyapatite compared to uncoated hydroxyapatite. Here, we determined the kinetics of HGF adsorption onto a dense HA surface and identified the influence of HGF coating on osteoblast gene expression [42].

Fibroblast growth factor: In vitro tests indicated that fibroblast growth factor (FGF)-2 was the most effective at encouraging cell proliferation; colonies produced under clonal circumstances were around 2.5 times larger in the presence of FGF-2 [43]. FGF-2 alone accelerated bone growth and facilitated osseointegration in implants with low primary stability, resulting in high stability [44].

Drug delivery coatings: Antibacterial dental implant coatings include molecules, metals, minerals, antibiotics, and antiseptics [45]. Percutaneous and transmucosal implants, including external fixing pins and dental implants, are particularly vulnerable to pathogenic microbial infection. Periodontal pathogens, e.g., *Staphylococcus aureus*, *Staphylococcus epidermidis*, and *Pseudomonas aeruginosa*, could get access during and after surgery, sticking to the implant's surface and causing infections [46]. These bioactive coatings transport pharmaceuticals like gentamycin, vancomycin, and amoxicillin in a controlled manner, requiring a vector employment. Calcium phosphates, which are biocompatible and osteoconductive, have been confirmed as potential vectors of bioactive chemicals [46]. Medication coatings applied to the surface of dental implants can include antibiotics, simvastatin, and bisphosphonates. Antimicrobial surfaces in dental implants have been produced using two main approaches. Type I surfaces actively release antimicrobial agents, limiting bacterial adhesion and increasing bacterial killing. In contrast, type II surfaces permanently attach antimicrobials to prevent long-term bacterial attachment and promote bacterial death [47].

Antimicrobial peptides: A previous study investigated the modified antimicrobial peptide (AMP) RRP9W4N's antibacterial effect on bacterial biofilms and its influence on osseointegration at the implant healing site. The peptide was incorporated into mesoporous titanium and then applied as a coating on titanium implants. In vitro testing indicated that the AMP preserved its antibacterial capabilities, while in vivo results showed no deleterious effects on osseointegration [48]. Peptide coatings were ideal for generating a peri-mucosal seal around dental implants [49].

Gentamicin coatings: Gentamicin coatings on implants can prevent implant failure by decreasing the chances of infection immediately after placement [50].

Peptide and Protein Functionalization

The application of proteins and peptides in implantology is not extensively documented. Comprehensive research consolidating relevant compounds into a single study would benefit clinicians and institutions involved in surgical implant placement [51].

Extracellular matrix (ECM) components: Ligament-anchored implants, or implants covered by periodontal tissue, are considered the gold standard in dentistry. This includes harnessing the activities of mesenchymal stem cells to drive the induced expression of osteoblast-related genes and release ECM components [52]. Bonnans et al. (2014) revealed that the ECM is a dynamic and continuously remodeled structure that regulates tissue homeostasis, with its components serving as potential therapeutic targets. As a result, coating dental implants with a biomimetic bone mixture combining calcium phosphate (CaP) and ECM components looks to be a promising method for increasing osseointegration [53]. During the proliferative stage of osseointegration, fibroblast growth factors stimulate the secretion of ECM proteins like hyaluronic acid, collagen, chondroitin sulfate, fibronectin, and elastin [47].

Collagen type 1: The early interaction between osteoblasts and implant surfaces is critical in osseointegration. The Ti6Al4V surface can be pre-coated with collagen type I to improve osseointegration [54]. Type I collagen was adsorbed or covalently immobilized on plasma-sprayed porous titanium surfaces. Surface characterization methods such as scanning electron microscopy, diffuse reflectance Fourier transform infrared spectroscopy (DR-FTIR), and X-ray photoelectron spectroscopy (XPS) showed that the titanium coatings were subject to biological modification. These methods proved the immobilizing effect of type I collagen on surfaces [55].

Biomimetic active peptide (P-15): Implants with high amounts of P-15 demonstrated a far more tremendous amount of bone-to-implant contact. Additionally, plants with high amounts of P-15 had significantly more substantial quantities of bone-to-implant contact [56].

Fibronectin-derived oligopeptide: F20, a fibronectin (FN)-derived oligopeptide, enhances osteoblast adhesion and proliferation. It stimulates osteogenesis via the Erk pathway and is an ideal biomolecule for the surface modification of dental implants to increase osseointegration [57].

Animal-inspired peptide: Animal-derived peptides are commonly used in biomedical applications because of their mechanical characteristics, biological compatibility, and chemical reactions. Peptides used in medical implants are generally obtained from or originate in microorganisms, including *Bombyx mori* and *Antheraea pernyi*, spiders such as *Nephila clavipes* and *Araneus diadematus*, and marine mussels [51,58].

Albumin: This protein coating can improve the biocompatibility of certain implants, such as bone transplants and sutures. Albumin is best recognized as an anti-attachment protein, but utilizing it on implantable surfaces has the opposite effect, i.e., it increases stem cell adherence and proliferation [59]. Albumin comprises 585 amino acids and has a low molecular weight of 66.5 kDa. It is a single-chain protein that is simple, non-glycosylated, and has hydrophobic areas and voids with no prosthetic groups present [60]. Proper sealing of the transmucosal soft tissue is essential for long-term implant durability. Hydrogenated titanium nanotubes (H2-TNTs) have been shown in studies to improve HGF adhesion to implant surfaces. Nanotubes are commonly employed as pharmaceutical carriers. Bovine serum albumin (BSA), the most common albumin in body fluid, is a standard-loading protein that plays a key part in cell adhesion [60].

Antibodies: Sclerostin, an osteocyte-expressed protein that regulates bone metabolism, inhibits Wnt signaling, limiting bone production. In preclinical trials comprising both maxilla and long bones, sclerostin inhibition by sclerostin antibody (SclAb) was demonstrated to improve bone quality and induce bone regeneration and osseointegration [61]. Sclerostin, a small glycoprotein, inhibits the WNT/β-catenin signaling pathway, leading to decreased bone growth. Sclerostin, which is produced chiefly by osteoblast lineage cells, inhibits bone formation and increases osteoclast activity. The concept is that antibodies (sclerostin) promote bone formation and decrease bone resorption by reducing sclerostin's interaction with WNT co-receptors, improving osteoblastic activity. Systemic ingestion of sclerostin antibodies has increased bone-implant contact, density of bone minerals, and cementogenesis [58].

Hormones: Three main hormones have been extensively researched to aid implant integration: parathyroid hormone (PTH), growth hormone (GH), and melatonin. PTH, released by the parathyroid glands, controls extracellular calcium and phosphate metabolism. It can promote bone growth by inhibiting sclerostin. Studies have shown that low, intermittent dosages of PTH have osteoanabolic effects, promoting osteoblast maturation and reducing osteoblast apoptosis, improving bone formation. Different research results demonstrate that intermittent injection of PTH is beneficial in stimulating new bone growth around implants [58].

This review gives an in-depth and complete analysis of how various surface coatings increase osseointegration in dental implants. Sandblasting is a standard method for treating dental implant (DI) surfaces to enhance osseointegration, and it has been clinically demonstrated to be beneficial [62]. Grit blasting improves the interlocking of mineralized bone and implant surfaces. Surface roughness at the nanoscale, obtained through micro-arc oxidation, considerably affects protein adsorption, osteoblastic cell adhesion, and, eventually, the rate of osseointegration [63]. Using lasers to change dental biomaterials' surfaces is a promising issue with vast potential for future research [64,65]. Plasma spray coatings may provide clinical benefits by enhancing biological fixation and osseointegration within the first six to 12 weeks following surgery, a critical healing period for implant-based arthrodesis operations [66].

Chemical Changes to Implant Surfaces

It has been established that anodization enhances surface characterization, which results in rougher titanium samples due to nanotube formation. This improves the surface energy and wettability, increasing osteoblast adhesion, fibronectin, and vitronectin cell expression [23]. Dental implants modified with a thin CaP coating improve osseointegration in healthy and osteoporotic patients [67]. Growth factors have shown exceptional osteogenic and angiogenic-inducing capabilities [29,68]. Animal research showed that the BMP group outperformed the non-BMP group. Chang et al. (2017) found no osteogenesis and lower mineralization in the non-BMP group after four weeks and revealed a modest increase in bone gain after four weeks [69]. Schorn et al. (2017) [70] and Chao et al. (2021) [71] discovered that maximum gain with BMP was obtained after 12 weeks and eight weeks, respectively, indicating the evaluation period for the influence of BMP. Both immunofluorescence and fluorescence-activated cell sorting (FACS) analysis demonstrated that anti-VEGF receptor (VEGFR) therapy significantly reduced CD31hiEMCNhi (a subtype of vessels in the murine skeletal system) vascular endothelial cells in the peri-implant bone. In addition, RNA sequencing transcriptional profiling showed that the production of several types of bone-forming factors, including bone morphogenetic protein 2 (BMP2) and fibroblast growth factor 2 (FGF2) in CD31hiEMCNhi endothelial cells derived from implanted tibiae, was reduced in anti-VEGFR-treated mice compared to controls [72]. Both immunofluorescence and FACS analysis revealed that anti-VEGFR therapy dramatically decreased CD31hiEMCNhi vascular endothelial cells in the peri-implant bone. In addition, RNA sequencing and transcriptional profiling showed that anti-VEGFR treatment reduced the level of expression of several types of osteogenic variables, such as BMP2 and FGF2, in CD31hiEMCNhi endothelial cell lines derived from implanted tibiae compared to controls [73].

Summary: Mechanism of Osseointegration

Some of these mechanisms are mentioned in the document, but let us go into more detail about the biological mechanisms by which implant surface alterations improve osseointegration.

Surface wettability: Chemical changes like anodization or hydroxylation provide hydrophilic surfaces, which are more receptive to biological fluids like blood and saliva. This increased wettability facilitates the first adsorption of proteins at a critical stage in osseointegration [22,74,75].

Adsorption of proteins: Protein adsorption from the surrounding biological fluids occurs immediately after implant placement in bone. Surface topography, especially roughness, influences the adsorbed protein layer's amount, shape, and composition. These proteins mediate cell attachment and impact subsequent cellular behavior, including vitronectin and fibronectin [76-78].

Cellular signaling pathways: Changes to the implant surface can cause some cells, such as osteoblasts, to activate cellular signaling routes. Surface roughness, for instance, can change the shape of cells and encourage the development of osteoblasts and bone. Growth factor coatings and other biological alterations directly introduce signaling molecules like BMPs, which initiate pathways that promote osteogenesis [79-82].

Critical analysis of this narrative review

The current study summarizes the literature on dental implant surface modification and its possible effect on osseointegration. Various research studies have been compiled to present an overview of different techniques and their documentary outcomes. It also discusses topographical modifications like sandblasting, acid etching, and laser treatment to alter the implant surface to enhance bone cell attachment. It also explains chemical modifications, e.g., anodization and calcium phosphate coatings, which aim to improve biocompatibility and accelerate bone formation. It also summarized the biological modification by growth factor coatings, including drug delivery systems designed to stimulate bone healing and prevent infections. The article also discusses future directions in implant surface modifications, including nanotechnology-based modifications, bioactive and drug-eluting coatings, 3D printing, plasma and ion-based techniques, biodegradable and innovative materials, and graphene and biomimetic coatings.

Future directions of research directives

Research on implant surface coatings is pursuing several novel approaches to improve osseointegration, reduce infections, and increase implant lifetime. Nanotechnology produces coatings and nanoscale roughness technologies that are antibacterial and encourage cellular adhesion. Bioactive and drug-eluting coatings that combine growth factors, antibiotics, or osteoinductive materials are being developed to promote healing and infection control. Additive manufacturing methods like 3D printing and surface patterning are being investigated to create tailored textures that maximize bone integration. Furthermore, methods based on plasma and ions, like ion implantation and plasma electrolytic oxidation (PEO), are being researched to improve biocompatibility and corrosion resistance. With the possibility of controlled disintegration and responsive drug release, biodegradable and innovative materials are increasingly gaining traction. Additionally, research is being done on graphene and biomimetic coatings to enhance mechanical strength, conductivity, and general biological compatibility, opening the door for implant technologies of the future.

Limitations of this narrative review

This review's limitation is its heavy reliance on animal studies. While animal research provides valuable insights, further human studies are needed to validate and translate these findings effectively into clinical applications and broader societal use.

## Conclusions

Modifying implant surfaces is essential for enhancing osseointegration, contributing to dental implants' long-term success. Topographical alterations, such as roughening and nanostructuring, improve mechanical interlocking and cellular adhesion. Chemical modifications, including surface coatings and bioactive treatments, enhance biocompatibility and osteoconductive properties. Biological enhancements, such as incorporating growth factors and bioactive molecules, also accelerate bone healing and integration. By combining these strategies, implant surfaces can be optimized to facilitate faster and more effective osseointegration, ultimately leading to better clinical outcomes.
